# Mortars with Crushed Lava Granulate for Repair of Damp Historical Buildings

**DOI:** 10.3390/ma12213557

**Published:** 2019-10-30

**Authors:** Zbyšek Pavlík, Jaroslav Pokorný, Milena Pavlíková, Lucie Zemanová, Martina Záleská, Martina Vyšvařil, Tomáš Žižlavský

**Affiliations:** 1 Department of Materials Engineering and Chemistry, Faculty of Civil Engineering, Czech Technical University in Prague, Thákurova 7, CZ-166 29 Prague, Czech Republic; jaroslav.pokorny@fsv.cvut.cz (J.P.); milena.pavlikova@fsv.cvut.cz (M.P.); lucie.zemanova@fsv.cvut.cz (L.Z.); martina.zaleska@fsv.cvut.cz (M.Z.); 2 Institute of Chemistry, Faculty of Civil Engineering, Brno University of Technology, Veveří 331/95; 602 00 Brno, Czech Republic; vysvaril.m@fce.vutbr.cz (M.V.); zizlavsky.t@fce.vutbr.cz (T.Ž.)

**Keywords:** repair mortars, compatibility, lava granulate, functional properties, hygrothermal performance

## Abstract

In this paper, crushed lava granulate was used as full silica sand replacement in composition of repair mortars based on hydrated lime, natural hydraulic lime, or cement-lime binder. Lava granules were analyzed by X-ray fluorescence analysis (XRF), X-ray diffraction (XRD), and scanning electron microscopy (SEM). Particle size distribution of both silica and lava aggregates was assessed using standard sieve analysis. Hygrothermal function of the developed lightweight materials was characterized by the measurement of complete set of hygric, thermal, and structural parameters of the hardened mortar samples that were tested for both 28 days and 90 days cured specimens. As the repair mortars must also meet requirements on mechanical performance, their compressive strength, flexural strength, and dynamic Young’s modulus were tested. The newly developed mortars composed of lava aggregate and hydrated lime or natural hydraulic lime met technical, functional, compatibility, and performance criteria on masonry and rendering materials, and were found well applicable for repair of historically valuable buildings.

## 1. Introduction

Historical structures plastered with valuable and highly decorative facades are an essential part of our cultural heritage. Various kinds of mortars and plasters have been utilized all over the world from ancient times to modern days. In this sense, over time, their composition and in particular binder components have changed, from clearly natural materials, often represented by clays [1,2,3], to traditional binders, such as air lime [4,5,6,7], natural hydraulic lime [8,9,10,11,12], and their combination with natural or/and artificial pozzolans (diatomaceous earth, tuffs, zeolite, pumice, etc.) [13,14,15]. Brick dust is also considered a traditional component of mortars since its usage in lime mortars dates from the Minoan period through the ancient Greek and Roman civilizations [16]. Based on materials and production technology available, until the 1850s, air-lime blended with natural pozzolans was the main binder for rendering and plastering purposes [17]. With industry development at the end of 19th century, the first artificial hydraulic lime and later Portland cement (PC) and its decorative derivation, white cement, appeared. These made more strengthened plastering layers to resist weathering, moisture induced corrosion, and freeze/thaw cycles [18]. Based on that, PC became the prevailing binder of the construction industry and thereby also for mortars production. On the other hand, remarkably preserved buildings and ruins of structures aged hundreds or even thousand of years with lime-based facades and plasters are well documented [19,20,21]. Authors also pointed out the important role of the binder and aggregate ratio, particle size distribution of used aggregate, and method of plaster application that related to final total open porosity of hardened mortar layers and their corresponding durability.

The main role of traditional plasters is to ensure a sufficient protection of masonry against environmental deterioration. Their visual effect, however, importantly influences the look of buildings [22]. In renewal of historical and older buildings, material ageing is considered a universal phenomenon [23]. Internal renders as well as external plasters are threatened by many agents, e.g., rising dampness, water soluble salts, weathering combined with temperature changes, and polluted environment effects [24,25]. Therefore, their resistance against these harmful factors is required in the period of use. Nevertheless, as many historical buildings suffer from environmental action and exhibit excessive damage, there is a need for refurbishment and repair of existing rendering and plastering layers. Nowadays, cultural heritage authorities and building engineers put emphasis on the use of compatible materials for repair work that must meet the functional requirements on their properties, durability, performance, and also conform to building standards. Currently, manufactured plastering materials are not compatible with those originally used in historical or old structures, whereas these do not respect historically applied materials [26]. On this account, there is a need to develop new mortars for plastering, rendering, and masonry purposes that will be compatible with materials originally inbuilt and for their properties to comply with present technical and functional standards. Due to several severe problems associated with the use of PC-based mortars in repair works [27,28,29,30,31], they are no longer applicable for rendering and plastering of historically valuable buildings and cultural heritage monuments. As PC has some inadequate properties and is incompatible with many traditional materials inbuilt in historical buildings [32,33,34,35], air lime, natural hydraulic lime, lime-pozzolan, and PC-lime-based mortars represent alternatives to PC mortars which are too rigid, low water vapor permeable, and susceptible to frost damage. Nowadays, lime-based mortars and their derivatives are considered by the cultural heritage community as the most suitable for repair and renewal of old buildings.

Requirements on reducing the energy consumption of newly constructed and old/historical buildings are increasing, in particular, in the connection with the goals of the European Union policies on energy and climate towards 2020, 2030, and 2050, which were adopted in order to achieve Paris Agreement targets [36]. Buildings are considered to be the biggest energy consuming sector worldwide, accounting for over one third of the total final energy consumption [37]. As over 60% of energy in the EU household sector is used for space heating [38], the refurbishment of existing buildings is necessary to achieve EU objectives. To decrease the energy consumption related to heating and cooling of existing buildings, one possible solution is to modify their envelopes. In rehabilitation of later and modern buildings, external thermal insulating composite systems (ETICSs) based on, for example, expanded polystyrene, mineral wool, or wooden wool boards, can be used. On the other hand, to improve thermal insulation parameters of historical buildings with valuable aesthetic facades, this practice is not applicable and such interventions are excluded. In this case, application of repair thermal insulation plasters looks like a promising solution. Of course, thermal insulation plasters are less effective than ETICSs, but they represent a simple and cost-effective alternative acceptable by both cultural heritage authorities and construction practice. There are several thermal insulation renders and plasters on the construction market, mostly based on cementitious binders or cement blends with several mineral admixtures. The most characteristic components of thermal insulation renders are light-weight aggregates and fibrous materials [39]. According to Barbero [40], thermal insulation plasters based on organic foamy materials and inorganic fibers represents 87% of the European market. The remaining 13% is made up from natural products, such as cork [41], hemp shive [42], palm date fibers [43], wood wool [44], flax and straw rape [45], etc. Also, inorganic expanded materials, such as perlite and expanded glass have found use in composition of thermal insulation mortars [46,47]. Incorporation of innovative high thermal insulating aggregates, e.g., aerogels, has also been studied but their presence on the market is still at a low level [48,49,50].

Because most of the commercially produced thermal insulation plasters does not meet requirements of cultural heritage authorities, new types of lightweight mortars for repair of historical buildings have been designed and tested in this study. As stated above, a general consensus accepted by the cultural heritage authorities and researchers working in the field of repairing historical valuable buildings is that the use of cement-based mortars is not a proper solution for buildings rendered and constructed from air lime- and natural hydraulic lime-based mortars. On the other hand, in buildings built from the end of the 19th century and in 20th century, PC became the prevailing binder. In these structures, the cement-based repair mortars can find use as well as cement-lime mortars and materials where part of the PC is replaced with pozzolanic admixtures. Therefore, the aim of the presented work was to use in the composition of mortars binders compatible with the original, e.g., hydrated lime, natural hydraulic lime, and PC-hydrated lime blend. As reported by Tchamdjoua [51], volcanic aggregates based on volcanic tuff can be advantageously used in mortars production. By the use of these types of aggregates, weight reduction, enhanced thermal insulation function, and improved workability can be achieved. As the use of volcanic aggregates dates back to the ancient times [52,53,54], applied lava granulate undoubtedly meets demands of the cultural heritage community on the use of natural, compatible, and functional materials in buildings repair. Moreover, the natural volcanic rock did not need any expensive and high energy demand processing compared, e.g., to the production of expanded clays-based aggregates. Based on a detailed review of published papers, no publication was found in scientific databases on the use of lava aggregates in mortars composition within the last 30 years, which is what clearly documents the novelty of the presented work. For newly developed mortars with lava aggregate, a unique set of structural, mechanical, hygric, and thermal properties was experimentally assessed, allowing assessment of the applicability of the developed mortars in repair and repointing of historical masonry and plastering.

## 2. Experimental

Hydrate lime- (HL), natural hydraulic lime- (NHL), and PC (Portland cement) -HL blend-based mortars were the subject of the extensive experimental campaign. In newly developed mortars, silica sand was fully replaced by crushed lava granulate. First, the raw materials used for mortars production were characterized in detail. Charaterization comprised assessment of their chemical and mineralogical composition, basic physical parameters, particle size distribution, and testing of lava pozzolanic activity and SEM morphology. The obtained data were then considered in mortar mix design. The casted mortar samples were cured for 28 days and 90 days, respectively. For the hardened mortar samples, the complete set of structural, mechanical, hygric, and thermal properties was experimentally assessed. Finally, the microstructure morphology of the fractured surface of the hardened samples was observed and analyzed using SEM.

### 2.1. Materials and Design

Commercial hydrated lime HL CL90-S (Čertovy Schody, Inc., Lhoist group, Tmaň, Czech Republic), natural hydraulic lime NHL 3.5 (Zement- und Kalkwerke Otterbein GmbH & Co. KG, Großenlüder, Germany), and laboratory prepared blend of HL CL90-S and PC CEM I 42.5 R (Českomoravský cement Inc., Radotín, Czech Republic) were used as binders in 3 prepared groups of mortar mixes. Each mortars group consisted of reference samples made of silica sand, and samples with crushed lava granulate fully replaced silica aggregate. The composition of mortar mixtures considered the constant binder/aggregate volume ratio of 1:1.15 based on a practical point of view, historical traditions, and results obtained by Lanas et al. [55]. Silica sand of particle size fraction 0–2 mm (Filtrační písky s.r.o., Chlum u Doks, Czech Republic) was mixed from three normalized sand fractions 0–0.5 mm, 0.5–1 mm, and 1–2 mm. The weight ratio of the particular sand fractions was 1:1:1. Lava granulate, the fine fraction 0/2 mm, came from Der Naturstein Garten, Hillscheid, Germany. The dosage of batch water was adjusted to get similar consistency of all studied mortars. Based on years of practical experience, the mortar mixtures were prepared using the correct amount of water required to obtain normal consistency and a good workability of the mortars (160 ± 5 mm; measured by the flow table test [56]). This consistency of lime mortars enables their easy application during rendering and good adhesion to the substrate. The composition of tested mortars is given in Table 1, where HL-R stands for a reference lime mortar, HL-LA is a lime mortar with lava granulate, PCHL-R and PCHL-LA are cement-lime mortars with silica and lava aggregates, NHL-R is a natural hydraulic lime reference mortar, and NHL-LA is a natural hydraulic lime mortar with lava aggregate.

Mortar samples were prepared and tested according to European standards EN 10115-2 [57] and EN 196-1 [58]. The test specimens were standard prisms 40 mm × 40 mm × 160 mm, and circular plate specimens having 30 mm thickness and diameter of 120 mm. The freshly casted samples were covered by PE foil and sprayed for 2 days by water in order to avoid their cracking, and after demolding stored for 26 days at a highly humid environment RH (95 ± 5%) at a temperature (22 ± 2 °C). Then, they were stored freely in a laboratory. In this way, high relative humidity favored the pozzolanic reaction and the hydration of anhydrous calcium silicates. On the other hand, curing at laboratory conditions allowed carbonation of hydrated lime mortars. The experimental tests were conducted for 28 days and 90 days hardened samples.

### 2.2. Binders Characterization

Among the basic physical characterization of HL, NHL, and PC, their loose bulk density, specific density, specific surface, and particle size distribution were assessed. The loose bulk density was obtained on a gravimetric principle, i.e., from the measurement of dry sample mass and its volume. The specific density was determined on the helium pycnometry principle using Pycnomatic ATC (Thermo Scientific, Milan, Italy). The particle size distribution was analyzed by an Analysette 22 Micro Tec plus (Fritsch, Idar-Oberstein, Germany) working on a laser diffraction principle. The Blaine specific surface was measured in accordance with the EN 196-6 [59].

For the measurement of chemical composition of applied binders, an ARL QUANT´X EDXRF Spectrometer (Thermo Scientific, Madison, WI, USA), equipped with a Rh X-Ray tube and Si(Li) detector crystal was used. The resulting data were collected and evaluated by the UniQuant ED 6.32 software (Thermo Scientific, West Palm Beach, FL, USA). The relative accuracy was 0.5% to 5.0%, depending on the standards quantity and concentration of analyzed substances. 

X-ray diffraction (XRD) data were collected by PANalytical Empyrean XRD (Malvern Panalytical Ltd., Royston, UK) with Cu-Kα as the radiation source (λ = 1.540598 Å for Kα1, accelerating voltage 45 kV, beam current 40 mA, diffraction angle 2θ in the range from 5° to 80° with a step scan of 0.01°. Data evaluation was performed by HighScore Plus software 4.8 (Malvern Panalytical Ltd., Royston, UK) version 3.0 using ICDD and ICSD databases.

The basic physical properties and particles size distribution parameters of applied binders are given in Table 2. The highest Blaine fineness exhibited HL, the lowest PC, and that of NHL was in the middle. This was in agreement with the loose bulk density and specific density data. The measurement of particle size distribution revealed that NHL was coarser compared to HL and PC, but still sufficient for binding purposes.

The binders’ chemical and phase composition measured by XRF and XRD analyses are shown in Table 3. It complies with the nature of analyzed binders. The main constituent of HL was CaO coming from calcination of calcite. NHL was formed mainly from CaO, hydraulic oxides were present in a limited extent, only similarly as reported by Luo et al. [60]. Main oxides contained in PC were SiO_2_, Al_2_O_3_, Fe_2_O_3_, and CaO, which are typical constituents of Portland clinker [61]. NHL and PC contained significant amounts of amorphous phases. On the other hand, HL was fully crystalline. In HL, the main crystalline phase was Portlandite. In NHL, the main mineralogical phases present were Larnite, Calcite, and Portlandite. On similar NHL phase composition reported, e.g., Grilo et al. [62]. The major mineral phases in PC were Alite, Aluminate, Larnite, and Brownmillerite [61].

### 2.3. Aggregates Testing

For silica and lava sand, basic physical parameters (loose bulk density, specific density) and chemical composition were measured using similar methods applied for binder analyses (see Section 2.2). As the laser diffraction method working device is not designed for measurement of coarser particles, the aggregate grain size analysis was performed using the standard sieve method according to the EN 933-1 [63] with sieves of the following mesh dimensions: 0.063; 0.125; 0.25; 0.5; 1.0; 2.0 mm. The water absorption was measured for samples immersed for 24 h in water as prescribed in the EN 1097-6 [64]. Moreover, pozzolanic activity of lava fine fraction was tested. The modified Chapelle method was used for assessment of the pozzolanic activity according to the standard NF P 18-513 [65]. The test is based on the reaction of 1 g of tested material in powder form with 2 g CaO in 0.25 L of water. The results, which are expressed in mg Ca(OH)_2_ fixed by 1 g of sample [66], give the straight information about the pozzolanic activity [67]. The morphology of lava particles was investigated using scanning electron microscope (SEM) Tescan Mira3 (TESCAN Brno, Ltd., Brno, Czech Republic). Elemental composition and mapping were performed using an energy dispersive spectroscopy (EDS) analyzer with XFlash 6/10 detector (Bruker AXS GmbH, Karlsruhe, Germany) and Quantax 400 software (Bruker AXS GmbH, Karlsruhe, Germany).

The loose bulk density and specific density of both used aggregates are introduced together with information on pozzolanic activity in Table 4. The ratio of loose bulk density and specific density, which was 0.63 for silica sand and 0.46 for lava granulate, gave information on lava porosity which was important for lightening of the mortar matrix and improvement of other related functional parameters, such as hygric and thermal. On lava porosity, evidence also resulted in water absorption that was 1.57% for silica sand and 8.73% for lava sand.

**Figure 1:**
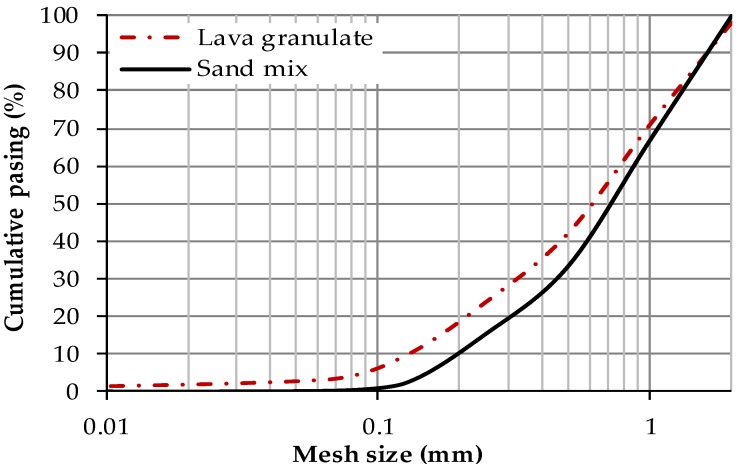
Particle size distribution of silica and lava aggregate.

Particle size distribution of silica and lava aggregates is graphed in Figure 1. Lava aggregate contained higher amounts of smaller particles compared to silica sand. Nevertheless, both types of aggregates had particle size in the 0–2 mm range.

The Chapelle test showed fixation of 746 mg Ca(OH)_2_/g of lava aggregate fine fraction (particle size <0.063 mm), which proved its reactivity in lime- and cement-based blends. According to Raverdy et al. [68,69,70,71,72,73,74], material is considered as pozzolana active if its Chapelle reactivity is ≥650 mg of Ca(OH)_2_/g of material. This condition was safely fulfilled. 

The chemical and phase composition of silica and lava sand measured by EDXRF spectrometer and XRD analysis is presented in Table 5. In lava sand, SiO_2_ and Al_2_O_3_ prevailed, which is in compliance with the Chapelle test result. According to the total alkali-silica (TAS) classification diagram (dependence of (Na_2_O + K_2_O) wt.% content on SiO_2_ wt.% content) for volcanic rocks, the applied lava sand belongs to ultrabasic basaltic rocks, namely, tephrites [69]. As reported, e.g., by Wakizaka [70] and Jozwiak-Niedzwiedzka [71], igneous rocks containing volcanic glass can be potentially dangerous in terms of alkali silica reaction (ASR). However, it was not this case, because alkali volcanic rocks-based aggregates are deleterious in terms of ASR, as their chemical composition expressed in wt.% exhibits the following chemical character: SiO_2_ > 50%, K_2_O > 3%, CaO < 5%, and MgO < 3% [72]. Silica sand was fully crystalline. In lava sand, a high amount of silica and alumina amorphous phases was identified, which clearly explained its pozzolanic activity. 

**Figure 2:**
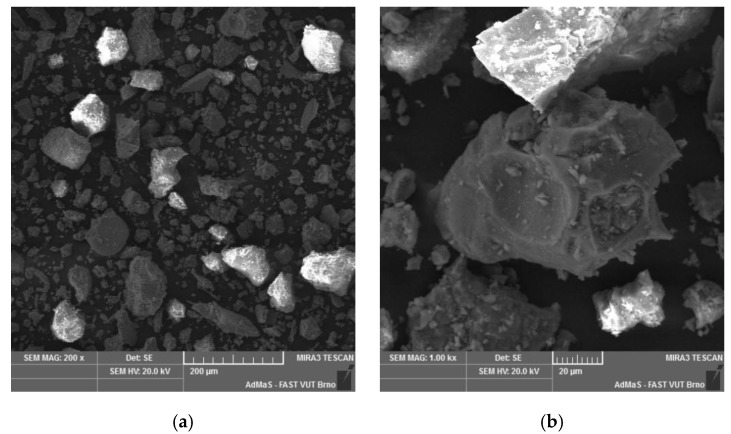
Lava morphology taken by SEM. (**a**) magnification 200×; (**b**) magnification 1000×.

The morphology of lava particles analyzed using SEM is apparent from Figure 2. The lava aggregate is made up of crushed volcanic rock particles of different shape, particle size, and angular edges. The high magnification made it possible to observe rugged morphology of lava particles with irregular/uneven sub-conchoidal fracture.

**Figure 3:**
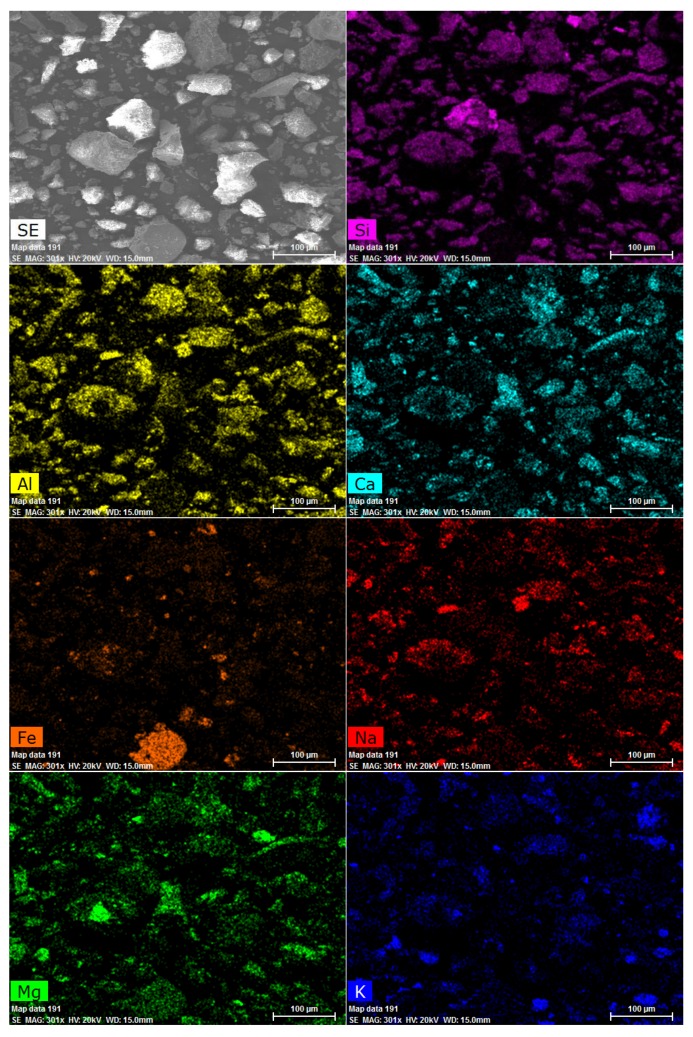
Lava elemental distribution maps.

The distribution of particular elements forming lava particles obtained by EDS is shown in Figure 3. No clustering was observed, i.e., all the analyzed elements in the studied sample were homogenously distributed.

### 2.4. Methods for Testing Hardened Mortar Samples

The measurement of hardened mortar specimens was conducted for 28 days and 90 days for the matured samples. 

The basic physical and structural characterization of hardened materials was done using the measurement of bulk density, specific density, and open porosity. In these tests, 5 samples that were vacuum dried at 60 °C were examined. The specific density *ρ_s_* (kg/m^3^) was obtained by helium pycnometry similarly as in the binders’ characterization (see Section 2.2). The dry bulk density *ρ_b_* (kg/m^3^) was determined in accordance with the EN 1015-10 [73]. The expanded combined uncertainty of the bulk density test was 1.4%. The total open porosity *ψ* (%) was calculated based on the bulk density and specific density tests results as formulated in Equation (1):

(1) $$\psi =100\left(1-\frac{\rho_{b}}{\rho_{s}}\right).$$

The expanded combined uncertainty of the total open porosity test was 2.0%.

Among the mortars’ mechanical parameters, dynamic Young’s modulus, flexural strength, and compressive strength were measured. For the particular mortar mixes, 5 prisms having size 40 mm × 40 mm × 160 mm were tested. First, the dynamic Young’s modulus *E_d_* (GPa) was measured using a DIO 562 apparatus (Starmans Electronic, Prague, Czech Republic) working on the frequency of 50 kHz. The ultrasonic pulse method was based on measurement of the travel time of the ultrasonic wave passing through the material. The relationship between *E_d_* (GPa), wave velocity *ν* (m/s), and bulk density *ρ_b_* (kg/m^3^) is given by Equation (2):

(2) $$E_{d}=\rho_{b}\nu^{2}.$$

As the moisture presence significantly affects propagation of ultrasonic waves, the tested specimens were dried at 60 °C in a vacuum dry kiln VACUCELL 22 ECO line (BMT Medical Technology Ltd., Brno, Czech Republic). The expanded combined uncertainty of this test was 1.7%.

Both strength tests were conducted in accordance with European standard EN 1015-11 [74]. Three-point bending strength test arrangement with a 100 mm span was used in the flexural strength *f_f_* (MPa) testing. On the fragments of broken specimens from the flexural strength test, the compressive strength *f_c_* (MPa) was assessed. The loading in the compressive strength test was 40 mm × 40 mm. The relative expanded uncertainty of these strength tests was 1.4%.

The ability of the studied mortar to absorb liquid water was described by the measurement of the water absorption coefficient and apparent moisture diffusivity. The free water intake experiment conducted in accordance with EN 1015-18 [75] was used for the determination of the water absorption coefficient *A_w_* (kg/(m^2^∙s^1/2^)) and capillary water content *w_cap_* (kg/m^3^). The test was performed in an automatic arrangement, i.e., changes in specimen mass were continuously recorded and the contact of the sample surface with water reservoir was not interrupted throughout the test. Using the original procedure proposed by Kumaran [76], the apparent moisture diffusivity *κ_app_* (m^2^/s) was calculated [77]. The expanded combined uncertainty of the water absorption test was 2.3%, and that of the apparent moisture diffusivity test was 3.5%.

For repair mortars, the ability to transport water vapor is of the particular importance. The water vapor transmission in tested materials was characterized by the measurement of the water vapor diffusion coefficient *D* (m^2^/s), water vapor resistance factor *μ* (−), and water vapor permeability *δ* (s). These parameters were tested using the cup method following the standard ISO 12572 [78]. Both the dry-cup and wet-cup arrangements of the test were applied. For the cup tests, circular plater specimens of 30 mm thickness and diameter of 120 mm were used. The 28-days samples placed in alumina cups were cured in a climatic chamber CLIMACELL 111 (BMT Medical Technology Ltd., Brno, Czech Republic) at (23 ± 0.5 °C) and RH (50 ± 5%) until their constant mass was reached. In the dry cup test, the cup contained silica gel, i.e., the relative humidity ratio simulated under and above the specimen was 2%/50% RH. In the wet-cup experiment, the humidity ratio was 93%/50% RH and the high RH inside the cup was obtained by saturated solution of KNO_3_. For each tested mortar, 5 specimens in the dry-cup test and 5 specimens in the wet-cup test were examined. The expanded combined uncertainty of the water vapor diffusion test was 2.0% for *D*, 2.8% for *μ*, and 2.1% for *δ*. 

As anticipated, the use of lava granulate in mix composition should improve mortars’ thermal properties. The investigated thermal parameters, the thermal conductivity *λ* W/(m∙K), and volumetric heat capacity *C_v_* (J/(m^3^∙K)), were tested in the dependence on moisture content, from the dry state to the fully water saturated state. These tests were done at laboratory temperature (23 ± 2 °C) using an ISOMET 2114 device (Applied Precision, Ltd., Bratislava, Slovakia). ISOMET 2114 operates on a transient technique based on recording temperature changes induced by heat impulse generated by the sensor [79]. The measurement accuracy was 5% of reading + 0.001 W/(m∙K) for the thermal conductivity in the range 0.015–0.70 W/(m∙K), and 10% of reading for the thermal conductivity ranging from 0.70 W/(m∙K) to 6.0 W/(m∙K). The accuracy of the volumetric heat capacity was 15% of reading +1 × 10^3^ J/(m^3^∙K). For each mortar type, 3 cubic samples having size of 70 mm matured for 90 days were tested using circular surface probe IPS 1105 (Applied Precision Ltd., Bratislava, Slovakia).

The microstructure morphology of fractured surface of mortar samples was determined on 90-days samples using scanning electron microscope (SEM) Tescan Mira3 (TESCAN Brno, Ltd., Brno, Czech Republic).

## 3. Results and Discussion

The basic structural parameters of tested mortars cured for 28 days and 90 days are summarized in Table 6. The porosity, which was considered the main parameter affecting overall mortars performance, decreased slightly with curing time. It was due to the continuous hydration and carbonation. Nevertheless, these differences in the porosity values and thus in the bulk density and specific density, in time, can be considered as inconspicuous and have no practical sense for mortars use. On the other hand, substitution of silica sand with lava aggregate markedly increased the porosity of all examined mortars which was good for their presumed application in repair of historical damp masonry and facades. The increase in porosity of 28-days samples was approximately 27% for HL-LA, 21% for PCHL-LA, and 27% for NHL-LA. The increase in porosity was assigned to porosity of lava particles, their irregular shape, and higher amount of batch water in mixes with lava aggregate. Accordingly, the bulk density values were for all mortars with lava aggregate lower compared to that of corresponding reference mortars. It clearly demonstrated lightening of mortar structure by the use of lava aggregate.

Looking at the obtained data from the point of view of the effect of applied binders, the lowest porosity values were obtained for mortars on the basis of the PC/HL blend. The use of HL and NHL gave almost similar porosity, which was about 15% higher in the case of reference mortars and about 20% higher in the case of mortars with lava aggregate compared to values obtained for PCHL-R and PCHL-LA, respectively. Similar porosity values of NHL reference mortars were obtained recently, e.g., Garijo et al. [80]. Accordingly, Pachta et al. [81] received lime mortar with sand of siliceous origin porosity of 31%, which was in agreement with porosity value of control lime mortar. Palomar at al. [82] measured cement-lime mortars porosity of 25.6%. It was close to the porosity of PCHL-R.

**Figure 4:**
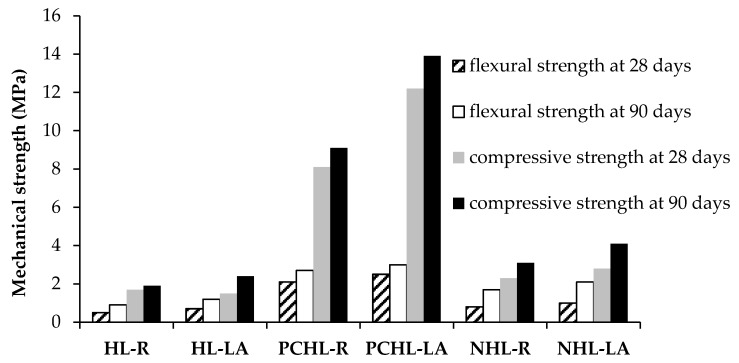
Mechanical parameters of tested mortars in dependence on curing time.

The mechanical parameters of the hardened mortars are summarized in Table 7 and Table 8. In addition, the strength data is for the sake of comparison graphed in Figure 4. The values of the tested mechanical characteristics of the control materials corresponded with the porosity data and with the character of applied binders. The mortars with lava sand had, for all studied mixes, higher mechanical resistance than reference materials. This was due to the rigid matrix of lava particles having higher mechanical strength compared to silica sand. Also, the pozzolanic activity of the lava fine particles contributed to the total mechanical resistance of lava-based mortars.

The highest mechanical resistance yielded cement-lime mortar, both reference and lava modified mixes. This was mainly due to the high content of PC in the blended binder. According to Ramesh et al. [83], the open porosity of cement-lime mortar increases with the lime dosage in the binder. In this way, the parameters of mortars can be simply modified. In literature, the values of Young’s modulus of cement-lime mortars were found to be significantly different, ranging from 3 GPa to 24 GPa [84]. In this sense, the *E_d_* values obtained for PCHL-R and PCHL-LA were approximately in the middle of this interval.

Unfortunately, as stated above, the use of PC in repair of historically valuable buildings is mostly forbidden by cultural heritage authorities. One of the reasons is mechanical incompatibility of the cement-based materials with those original [84] as rendering and masonry mortars should be considerably weaker than the old masonry to accommodate slight movements of the buildings and their structural elements. From this point of view, moderate mechanical resistance of NHL-based mortar is highly promising for its intended use in repair works. Building practice classifies mortars based on their 28-days compressive strength according to the European standard EN 998-1 [85]. In this respect, mortar HL-LA can be classified into category CS I (*f_c_* in the range 0.4–2.5 MPa), material NHL-LA into category CS II (*f_c_* in the range 1.5–5.0 MPa), and mortar PCHL-LA ranks into class CS IV (*f_c_* > 6.0 MPa). On the other hand, this standard does not pose any exact requirement on mechanical resistance of repair mortars. In literature, different requirements on the minimum mechanical resistance of mortars for repair or replacement of traditional lime-based rendering mortars are reported. In comprehensive review of the design and behavior of traditional lime-based renders, Nogueira et al. [86] recommended the minimum compressive strength from 0.4 to 2.5 MPa, and the minimum flexural strange in the range 0.2–0.7 MPa. In this respect, lime-based mortar with lava aggregate was acceptable for repair purposes, and NHL-based mortar even exceeded the minimum demands on mechanical resistance. 

The parameters characterizing the ingress of liquid water into studied mortars are introduced in Table 9. The use of lava granulate led mostly to the slight decrease in the water absorption coefficient. This can be assigned to the changes in mortars pore size distribution by the use of lava granulate. European standard EN 998-1 [85] requires repair mortars capillary water absorption > 0.30 kg/(m^2^∙s^1/2^), which met mortars based on HL and NHL. According to the standard EN 998-1 [85], HL- NHL-based mortars were categorized into class W I (*A_w_* ≤ 0.4 kg/(m^2^∙s^1/2^). Quantitatively similar moisture absorption observed, e.g., Pavlíková et al. 2019 [87] who obtained lime-based repair mortar *A_w_* = 0.32 kg/(m^2^∙s1^/2^) [84]. Also, Fusade et al. [88] obtained NHL mortars water absorption coefficient in the range 0.30–0.379 kg/(m^2^∙s1^/2^).

The lower porosity of cement-lime mortar and the structure of PC hydrates were the cause of low water absorption. Therefore, PCHL-R and PCHL-LA mortars were found to be not suitable materials for repair of damp masonry, where high water absorption in time is the positive effect, as it would enable the mortar to better absorb moisture from the surrounding masonry and help it dry out as stated by Fucade et al. [88].

As the capillary water content increased with lava aggregate use, the apparent moisture diffusivity decreased. This is an interesting feature, pointing to the presence of bigger pores in mortars with lava that allowed high water absorption, but the gravity effect decelerated the moisture ingress. In these pores, capillary activity competed with gravity force.

The data on water vapor transmission parameters obtained for 28-days cured samples are given in Table 10. As reported in other studies [87,89,90], water vapor transmission, in the case of the wet-cup test, was slightly faster compared to dry-cup test results. This can be attributed to the filling of pores with water molecules, which reduced binding forces between water vapor molecules and surface of the pores that decelerate water vapor transport in the case of dry materials. The water vapor resistance factor of repair mortars for external elements must be ≤15, which is the limit required by EN 988-1 [85]. This demand safely met mortars based on HL and NHL. Thus, the encouraging results documenting high water vapor permeability of these materials can be from the point of view of possible water evaporation from damp laden masonry recommended for restoration projects. Similar results of water vapor transmission properties measured, e.g., Silva et al. [91] who analyzed the influence of natural hydraulic lime content on the properties of aerial lime-based mortars. For lime-based mortar, the authors obtained porosity of approximately 26% and *δ* = 1.47 × 10^−11^ s, for mortars made of lime and NHL blends, *δ* = 1.49–1.38 × 10^−11^ s. Accordingly, Bianco at al. [92] determined that for mortar composed of NHL as binder, with the addition of 2 wt.% of metakaolin *μ* = 10.

On the contrary, cement-lime mortars exhibited a much higher water vapor resistance factor than required by European standard. It is not surprising, as cement mortars and highly hydraulic mortars have excessive hardness, stiffness, and are impermeable for water in both liquid and gaseous forms [93]. On this account, as their use in repair of historical buildings would have adverse effects, their use in restoration application should be avoided.

**Figure 5:**
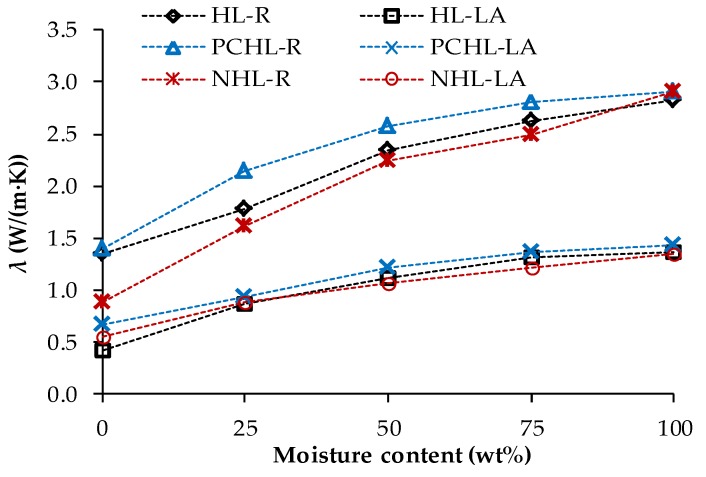
Thermal conductivity in dependence on moisture content—90-days samples.

**Figure 6:**
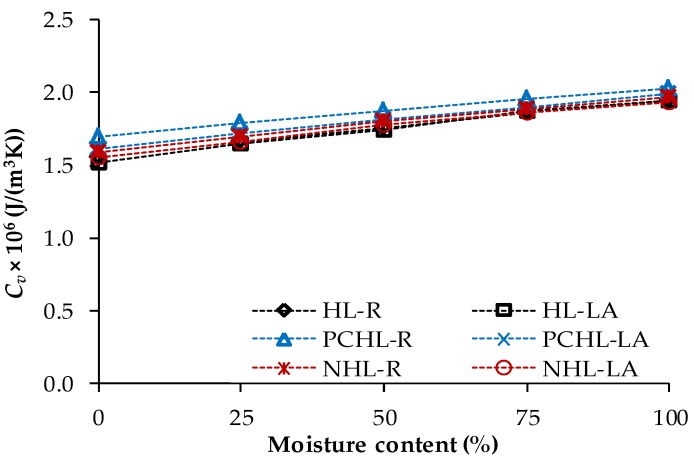
Moisture influenced volumetric heat capacity—90-days samples.

Heat transport and storage parameters acquired for 90-days samples in dependence on moisture content are shown in Figure 5 and Figure 6. As confirmed by many researchers in recent decades, the thermal properties of the porous construction materials are significantly affected by moisture content [49,94,95]. This is due to the high thermal conductivity of water (*λ_w_* = 0.56 W/(m∙K) at 20 °C) compared to dry air (*λ_a_* = 0.025 W/(m∙K) at 20 °C) [96] that fills the materials pores. If only a part of the pore space is filled by water, the cavities contain humid air having different thermal characteristics than the dry air.

The use of lava aggregate resulted in a considerable decrease in the thermal conductivity values compared to those of control materials. For the dry thermal conductivity, the decreases were 69% for HL-LA, 53% for PCHL-LA, and 38% for NHL-LA. This can be attributed to the higher porosity of these materials and also to the lava inner porosity itself. In accordance with the EN 998-1 [85] and the EN 1745 [97], the thermal conductivity of examined mortars met criteria for repair mortars. On similar thermal conductivity of lightweight mortar reported, e.g., Palomar and Barluenga [98] who measured cement-lime mortar with perlite and silica sand *λ* = 0.44 W/(m∙K).

The newly developed mortars with lava granulated exhibited slightly lower dependence of the thermal conductivity on moisture despite their high porosity. This feature is advantageous for practical use in repair of historical masonry that usually suffers from excessive moisture presence, and the repair materials usually serve as substrate for moisture transport from inner parts of masonry and consequently enable water evaporation. In this case, the low dependence of the thermal conductivity on moisture content allows to maintain moderate thermal insulation function even in moisture presence.

Taking into consideration the measuring uncertainty of the volumetric heat capacity test, the observed differences in *C_v_* values can be accounted as negligible, i.e., the replacement of silica sand with lava aggregate had no distinct effect on the heat storage capacity of the examined mortars. The dependence of the heat storage on the moisture presence in mortar samples was not significant and apparent as in the case of moisture dependent thermal conductivity. This was due to the volumetric heat capacity of water that is only about 3 times higher compared to the volumetric heat capacity of dry mortar samples.

**Figure 7:**
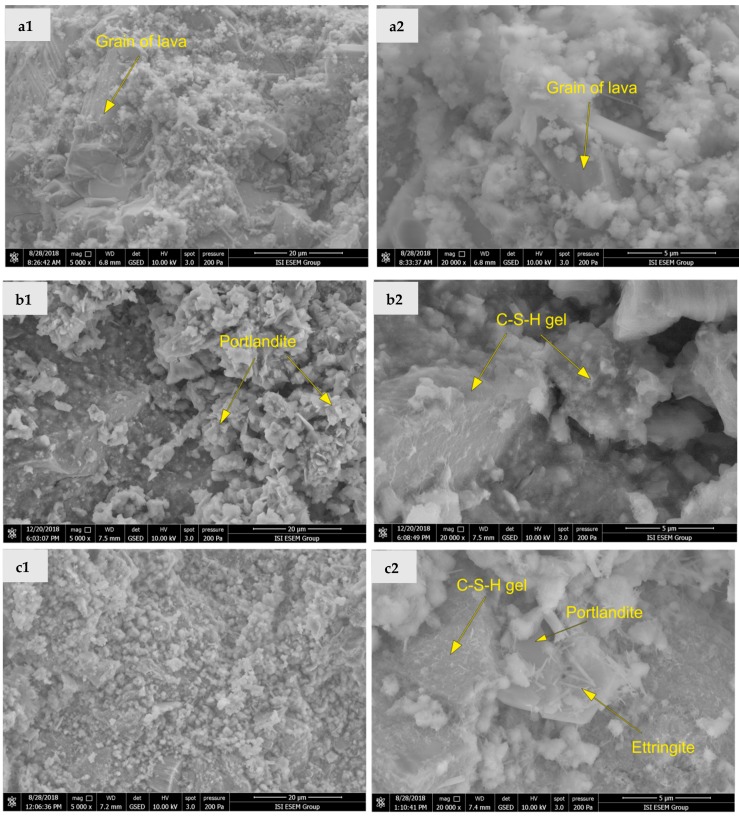
Microstructure of mortars with lava granulate (**a1**) HL-LA, magnification 5000×, (**a2**) HL-LA, magnification 20,000×; (**b1**) NHL-LA, magnification 5000×, (**b2**) NHL-LA, magnification 20,000×; (**c1**) PCHL-LA, magnification 5000×, (**c2**) PCHL-LA, magnification 20,000×.

The microstructure morphology of the fractured surface of mortar samples determined for 90-days samples using SEM is shown in Figure 7a–c. The SEM analysis allowed identification of lava grains covered and interconnected with Portlandite, CSH-gels, and in the case of PCHL-LA also with Ettringite. At first sight, the microstructure of the particular studied mortars differed. Mortar PCHL-LA had, in comparison with the other two mortars, a denser and more compact structure, which was in agreement with its lowest porosity and binder type. On the other hand, pores having size in the μm range were observed in materials HL-LA and NHL-LA. It was evident that the highly porous microstructure of HL- and NHL- based mortars was the reason behind their high water absorption, high water vapor permeability, and low thermal conductivity.

## 4. Conclusions

Lava granulate was studied as a possible full replacement of silica sand in the composition of lime-, cement-lime-, and natural hydraulic lime-based mortars. In order to achieve similar workability as the reference mortar mixes with silica sand, the batch water dosage was adjusted due to the higher water absorption of lava particles in the comparison with silica sand. The use of lava sand as an aggregate greatly increased the open porosity of the developed mortars. All the mortars with incorporated lava aggregate exhibited sufficient mechanical resistance in strength classes CS I (HL-LA), CS II (NHL-LA), CS IV (PCHL-LA), and the proved pozzolanic activity of lava fine particles contributed to the total mechanical strength. Based on the tests of mechanical properties, the mortars on the basis of cement/hydrated lime blend were found to be too rigid for repair of historical masonry. These mortars also exhibited low permeability for water in both liquid and gaseous phases. However, they can be safely used in construction of contemporary buildings. On the other hand, mortars based on hydrated lime and natural hydraulic lime met the technical requirements imposed by the European standards on thermal and hygric performance of repair mortars. Therefore, they were found compatible with materials of historical masonry, and well applicable for their repair. These materials possess high water absorption capacity and low water vapor resistance that ensure possible drying of damp laden masonry which is often the case of historical and older buildings. In this sense, the interconnectivity between mortar and substrate will be of the particular importance. The lava-containing mortars had considerably lower thermal conductivity than mortars with silica sand. Also, the thermal conductivity dependence on moisture content was reduced by the use of lava aggregate. This pointed out to their possible application in moderation of thermal performance of moist historical buildings as they can find use for the rendering, repointing, and walling purposes. In summary, it can be concluded, that the lava granulate is an effective and convenient aggregate for lime- and natural hydraulic lime-based mortars, especially in regions where lava resources are abundant.

## Figures and Tables

**Table 1:** Composition of studied mortars (kg/m^3^).

Mortar	Hydrated Lime	Portland Cement	Natural Hydraulic Lime	Sand Mix	Lava Granulate	Water
HL-R	326.1	-	-	1303.5	-	391.3
HL-LA	380.2	-	-	-	1292.6	437.4
PCHL-R	241.9	241.9	-	1354.8	-	348.3
PCHL-LA	272.7	272.7	-	-	1309.1	391.8
NHL-R	-	-	410.0	1394.7	-	307.7
NHL-LA	-	-	482.5	-	1385	386.0

**Table 2:** Physical properties and particle size distribution of HL, NHL and PC.

Material	Specific Surface (m^2^/kg)	Loose Bulk Density (kg/m^3^)	Specific Density (kgm^3^)	d_10_	d_50_	d_90_
				(µm)		
HL	2211	233	2210	0.8	4.2	50.3
NHL	1090	671	2590	23.5	52.4	69.1
PC	360	968	3129	6.0	22.8	32.4

**Table 3:** Chemical and phase composition of applied aerial and hydraulic binders (wt.%).

Oxides Composition	HL	NHL	PC
SiO_2_	0.2	6.7	20.2
Al_2_O_3_	0.1	3.7	4.9
Fe_2_O_3_	0.1	2.5	3.4
TiO_2_	-	0.2	0.4
CaO	98.7	84.3	65.3
MgO	0.4	1.9	1.5
K_2_O	-	0.5	0.9
Na_2_O	-	-	0.1
SO_3_	0.1	-	3.2
**Mineral**	**HL**	**NHL**	**PC**
Alite	-	-	50.6
Aluminate	-	2.7	3.9
Larnite	-	22.5	4.5
Brownmillerite	-	1.4	8.6
Brucite	0.5	-	-
Calcite	1.8	6.2	-
Gypsum	-	-	3.8
Portlandite	97.1	41.3	-
Amorphous phases	-	25.1	28.4

**Table 4:** Physical properties of used aggregates.

Material	Loose Bulk Density (kg/m^3^)	Specific Density (kg/m^3^)	Pozzolanic Activity (mg Ca(OH)_2_/g)
Silica sand	1670	2 647	21
Lava granulate	1410	3 060	746

**Table 5:** Chemical and phase composition of silica sand and lava granulate (wt.%).

Substance	Silica Sand	Lava Sand
SiO_2_	98.5	43.2
Al_2_O_3_	0.4	13.5
Fe_2_O_3_	0.2	10.7
TiO_2_	0.1	2.6
CaO	-	11.9
MgO	-	8.8
K_2_O	0.1	2.8
Na_2_O	-	3.8
SO_3_	-	0.1
P_2_O_5_	-	0.5
**Mineral**	**Silica Sand**	**Lava Sand**
Biotite	-	0.8
Clinopyroxene	-	17.0
Diopside	-	24.8
Hematite	-	5.7
Hornblende	-	1.5
Microcline	0.4	-
Leucite	-	9.9
Nepheline	-	9.7
Quartz	97.9	1.9
Sanidine		11.2
Staurolite	1.1	-
Amorphous phases	-	17.1

**Table 6:** Basic physical parameters of hardened mortars.

Mortar	Bulk Density (kg/m^3^)		Matrix Density (kg/m^3^)		Total Open Porosity (%)	
	Curing Period (days)					
	28	90	28	90	28	90
HL-R	1757	1783	2598	2611	32.4	31.7
HL-LA	1672	1695	2836	2773	41.0	38.9
PCHL-R	1815	1845	2525	2535	28.1	27.2
PCHL-LA	1798	1805	2719	2697	33.9	33.1
NHL-R	1781	1813	2587	2625	31.1	30.9
NHL-LA	1716	1756	2840	2798	39.6	37.3

**Table 7:** Flexural and compressive strength of tested mortars including standard deviation (SD).

Curing Period (days)								
Mortar	28		90		28		90	
	*f_f_* (MPa)	SD	*f_f_* (MPa)	SD	*f_c_* (MPa)	SD	*f_c_* (MPa)	SD
HL-R	0.5	0.03	0.9	0.05	1.7	0.06	1.9	0.06
HL-LA	0.7	0.03	1.2	0.04	1.5	0.05	2.4	0.05
PCHL-R	2.1	0.11	2.7	0.05	8.1	0.08	9.1	0.07
PCHL-LA	2.5	0.08	3.0	0.08	12.2	0.10	13.9	0.07
NHL-R	0.8	0.05	1.7	0.08	2.3	0.04	3.1	0.06
NHL-LA	1.0	0.05	2.1	0.07	2.8	0.06	4.1	0.08

**Table 8:** Dynamic Young’s modulus of tested mortars including standard deviation (SD).

Curing Period (days)				
Mortar	28		90	
	*E_d_*	SD	*E_d_*	SD
	(GPa)		(GPa)	
HL-R	2.7	0.05	2.9	0.07
HL-LA	3.2	0.03	3.9	0.06
PCHL-R	10.9	0.08	11.2	0.09
PCHL-LA	14.4	0.11	16.2	0.12
NHL-R	4.3	0.05	5.2	0.05
NHL-LA	5.2	0.06	8.3	0.07

**Table 9:** Liquid water transport parameters.

Mortar	*A_w_* (kg/(m^2^∙s^1/2^)		*w_cap_* (kg/m^3^)		*κ_app_* (m^2^/s)	
	Curing Period (days)					
	28	90	28	90	28	90
HL-R	0.36	0.34	249.7	245.0	2.08 × 10^−6^	1.96 × 10^−6^
HL-LA	0.37	0.32	271.3	267.6	1.86 × 10^−6^	1.43 × 10^−6^
PCHL-R	0.13	0.12	205.1	202.0	4.02 × 10^−7^	3.53 × 10^−7^
PCHL-LA	0.12	0.10	264.6	259.2	2.06 × 10^−7^	1.48 × 10^−7^
NHL-R	0.33	0.32	217.6	217.0	2.30 × 10^−6^	2.16 × 10^−6^
NHL-LA	0.32	0.30	272.1	266.0	1.38 × 10^−6^	1.27 × 10^−6^

**Table 10:** Water vapor transmission properties.

Dry-Cup				
Mortar	*δ* (×10^−11^ s)	*D* (×10^−6^ m^2^/s)	*μ* (−)	*μ* Difference from Reference (%)
HL-R	1.77	2.42	11.1	-
HL-LA	1.84	2.51	10.7	−3.6
PCHL-R	0.78	1.07	25.3	-
PCHL-LA	0.95	1.30	20.7	−18.1
NHL-R	1.59	2.17	12.4	-
NHL-LA	1.64	2.25	12.0	−3.2
**Wet-Cup**				
HL-R	1.87	2.56	10.5	-
HL-LA	1.94	2.65	10.2	2.9
PCHL-R	0.94	1.28	21.0	-
PCHL-LA	1.03	1.40	19.2	−8.6
NHL-R	1.84	2.52	10.7	-
NHL-LA	1.93	2.64	10.2	−4.7

## References

[1] Aubert J.E., Marcom A., Oliva P., Segui P. (2015). Chequered earth construction in south-western France. J. Cult. Herit..

[2] Aguliar R., Marques R., Boyer K., Martel C., Trujilano F., Boroschek R. (2015). Investigation on the structural behaviour of archaeological heritage in Peru: Form survey to seismic assessment. Eng. Struct..

[3] Gomes M.I., Goncalves T.D., Faria P. (2016). Hydric behavior of earth materials and their stabilization with cement or lime: Study on repair mortars for historical rammed earth structures. J. Mater. Civ. Eng..

[4] Ponce-Anton G., Arizzi A., Zuluaga M.C., Cultrone G., Ortega L.A., Mauleon J.A. (2019). Mineralogical, textural and physical characterization to determine deterioration susceptibility of Irulegi castle lime mortars (Navarre, Spain). Materials.

[5] Jonaitis B., Antonovic V., Sneideris A., Boris R., Zavalis R. (2019). Analysis of physical and mechanical properties of the mortar in the historic retaining wall of the Gediminas Castle Hill (Vilnius, Lithuania). Materials.

[6] Sutti M.L., de Aguiar M.O.S., Fioriti C.F., Christofani M.P.H. (2019). Characterization of historical coating mortars of La Ceramo factory in Valencia. Vitr. Int. J. Archit. Technol. Sustain..

[7] Guerra F.L., Lopes W., Cazarolli J.C., Lobato M., Masuero A.B., Dal Molin D.C.C., Bento F.M., Schrank A., Vainstein M.H. (2019). Biodeterioration of mortar coating in historical buildings: Microclimatic characterization, material, and fungal community. Build. Environ..

[8] Moropoulou A., Bakolas A., Anagnostopoulou S. (2005). Composite materials in ancient structures. Cem. Concr. Compos..

[9] Maravelaki-Kalaitzaki P., Bakolas A., Karatasios I., Kilikoglou V. (2005). Hydraulic lime mortars for the restoration of historic masonry in Crete. Cem. Concr. Res..

[10] Akcay C., Sayin B., Yildizlar B. (2017). The conservation and repair of historical masonry ruins based on laboratory analyses. Constr. Build. Mater..

[11] Tenconi M., Karatasios I., Bala’awi F., Kilikoglou V. (2018). Technological and microstructural characterization of mortars and plasters from the Roman site of Qasr Azraq, in Jordan. J. Cult. Herit..

[12] Callebaut K., Elsen J., Van Balen K., Viane W. (2001). Nineteenh century hydraulic restoration mortars in the Saint Michael’s Church (Leuven, Belgium) Natural hydraulic lime or cement?. Cem. Concr. Res..

[13] Columbu S., Garau A.M., Luglie C. (2019). Geochemical characterisation of pozzolanic obsidian glasses used in the ancient mortars of Nora Roman theatre (Sardinia, Italy): Provenance of raw materials and historical-archaeological implications. Arch. Anthropol. Sci..

[14] Papayianni I., Stefanidou M. (2006). Stremgth-porosity relationships in lime-pozzolan mortars. Constr. Build. Mater..

[15] Wang J., Zhao T. (2017). Regional energy-environmental performance and investment strategy for China’s non-ferrous metals industry: A non-radial DEA based analysis. J. Clean. Prod..

[16] Stefanidou M., Assael M., Antoniadis K., Matziaroglou G. (2010). Thermal conductivity of building materials employed in the preservation of traditional structures. Int. J. Thermophys..

[17] Gris E.R., Paine K.A., Heath A., Norman J., Pinder H. (2013). Compressive strength development of binary and ternary lime-pozzolan mortars. Mater. Des..

[18] Mounir S., Hamid K.A., Maaloufa Y. (2015). Thermal inertia for composite materials white cement-cork, cement mortar-cork, and plaster-cork. Energy Procedia.

[19] Singh M., Waghmare S., Kumar S.V. (2014). Characterization of lime plasters used in 16th century Mughal monument. J. Archaeol. Sci..

[20] Santos Silva A., Cruz T., Paiva M.J., Candeias A., Schiavon N., Mirão J.A.P. (2011). Mineralogical and chemical characterization of historical mortars from military fortifications in Lisbon harbor (Portugal). Environ. Earth Sci..

[21] Bochen J., Labus M. (2013). Study on physical and chemical properties of external lime-sand plasters of some historical buildings. Constr. Build. Mater..

[22] Mazhoud B., Collet F., Pretot S., Chamoin J. (2016). Hygric and thermal properties of hemp-lime plasters. Build. Environ..

[23] Bochen J. (2015). Weathering effects on physical-chemical properties of external plaster mortars exposed to different environments. Constr. Build. Mater..

[24] Borges C., Santos Silva A., Veiga R. (2014). Durability of ancient lime mortars in humid environment. Constr. Build. Mater..

[25] Groot C., van Hees R., Wijffels T. (2009). Selection of plasters and renders for salt laden masonry substrates. Constr. Build. Mater..

[26] Fassina V., Favaro M., Naccari A., Pigo M. (2002). Evaluation of compatibility and durability of a hydraulic lime-based plaster applied on brick wall masonry of historical buildings affected by rising damp phenomena. J. Cult. Herit..

[27] Sepulcre-Aguilar A., Hernández-Olivares F. (2010). Assessment of phase formation in lime-based mortars with added metakaolin, Portland cement and sepiolite, for grouting of historic masonry. Cem. Concr. Res..

[28] Mosquera M.J., Silva B., Prieto B., Ruiz-Herrera E. (2006). Addition of cement to lime based mortars: Effect on pore structure and vapor transport. Cem. Concr. Res..

[29] Faria-Rodrigues P., Henriques F.M.A. (2004). Current mortars in conservation: An overview. Restor. Build. Monum..

[30] Torney C., Forester A.M., Szadurski E.M. (2014). Specialist ‘restoration mortars’ for stone elements: A comparison of the physical properties of two stone repair materials. Herit. Sci..

[31] Pavlíková M., Zemanová L., Pokorný J., Záleská M., Jankovský O., Lojka M., Pavlík Z. (2019). Influence of Wood-Based Biomass Ash Admixing on the Structural, Mechanical, Hygric, and Thermal Properties of Air Lime Mortars. Materials.

[32] Elert K., Rodriguez-Navarro C., Pardo E.S., Hansen E., Cazalla O. (2002). Lime mortars for the conservation of historic buildings. Stud. Conserv..

[33] Ventolà L., Vendrell M., Giraldez P., Merino L. (2011). Traditional organic additives improve lime mortars: New old materials for restoration and building natural stone fabrics. Constr. Build. Mater..

[34] Silva B.A., Ferreira Pinto A.P., Gomes A. (2015). Natural hydraulic lime versus cement for blended lime mortars for restoration works. Constr. Build. Mater..

[35] Moropoulou A., Bakolas A., Moundoulas P., Aggelakopoulou E., Anagnostopoulou S. (2005). Strength development and lime reaction in mortars for repairing historic masonries. Cem. Concr. Res..

[36] Amanatidis G. European Policies on Climate and Energy towards 2020, 2030 and 2050. http://www.europarl.europa.eu/RegData/etudes/BRIE/2019/631047/IPOL_BRI(2019)631047_EN.pdf.

[37] Garcia-Saez I., Méndez J., Ortiz C., Loncar D., Becerra J.A., Chacartegui R. (2019). Energy and economic assessment of solar Organic Rankine Cycle for combined heat and power generation in residential applications. Renew. Energy.

[38] Intensity of Final Energy Consumption. https://www.eea.europa.eu/data-and-maps/indicators/final-energy-consumption-intensity-4/assessment-2.

[39] Giosuè C., Pierpaoli M., Mobili A., Ruello M.L., Tittarelli F. (2017). Influence of binders and lightweight aggregates on the properties of cementitious mortars: From traditional requirements to indoor air quality improvement. Materials.

[40] Barbero S., Dutto M., Ferrua C., Pereno A. (2014). Analysis on existent thermal insulating plasters towards innovative applications: Evaluation methodology for a real cost-performance comparison. Energy Build..

[41] Panesar D.K., Shindman B. (2012). The mechanical, transport and thermal properties of mortar and concrete containing waste cork. Cem. Concr. Compos..

[42] Rahim M., Douzane O., Tran Le A.D., Langlet T. (2016). Effect of the moisture and temperature on thermal properties of three bio-based materials. Constr. Build. Mater..

[43] Ben Mansour N., Boudjemaa A., Gherabli A., Kareche A., Boudenne A. (2014). Thermal and mechanical performance of natural mortar reinforced with date palm fibers for use as insulating materials in building. Energy Build..

[44] Taoukil D., El Bouardi A., Ajzoul T., Ezbakhe H. (2012). Effect of the incorporation of wood wool on thermos physical properties of sand mortars. KSCE J. Civ. Eng..

[45] Rahim M., Douzane O., Tran Le A.D., Promis G., Langlet T. (2016). Characterization and comparison of hygric properties of rape straw concrete and hemp concrete. Constr. Build. Mater..

[46] Pichor W., Kaminski A., Syoldra P., Frac M. (2019). Lightweight cement mortars with granulated foam glass and waste perlite addition. Adv. Civ. Eng..

[47] Fenoglio E., Fantucci S., Serra. V., Carbonaro C., Pollo R. (2018). Hygrothermal and environmental performance of a perlite-based insulating plaster for the energy retrofit of buildings. Energy Build..

[48] Ibrahim M., Wurtz E., Biwole P.H., Achard P., Sallee H. (2014). Hygrothermal performance of exterior walls covered with aerogel-based insulating rendering. Energy Build..

[49] Glória Gomes M., Flores-Colen I., Manga L.M., Soares A., de Brito J. (2017). The influence of moisture content on the thermal conductivity of external thermal mortars. Constr. Build. Mater..

[50] Al Zaidi A.K.A., Demirel B., Atis C.D. (2019). Effect of different storage methods on thermal and mechanical properties of mortar containing aerogel, fly ash and nano-silica. Constr. Build. Mater..

[51] Tchamdjoua W.H.J., Grigolettoc S., Michelec F., Courardc L., Abidia M.L., Cherradia T. (2017). An investigation on the use of coarse volcanic scoria as sand in Portland cement mortar. Case Stud. Constr. Mater..

[52] Jackson M.D., Ciancio Rossetto P., Kosso C.K., Buonfiglio M., Marra F. (2011). Building materials of the theatre of Marcellus, Rome. Archaeometry.

[53] Di Benedetto C., Graziano S.F., Guarino V., Rispoli C., Munzi P., Morra V., Cappelletti P. (2018). Romans’ established skills: Mortars from D46b mausoleum, Porta Mediana necropolis, Cuma (Naples). Mediter. Archaelogy Archaom..

[54] Marra F., Anzidei M., Benini A., D’Ambrosio E., Gaeta M., Ventura G., Cavallo A. (2016). Petro-chemical features and source areas of volcanic aggregates used in ancient Roman maritime concretes. J. Volcanol. Geoth. Res..

[55] Lanas J., Alvarez-Galindo J.I. (2003). Masonry repair lime-based mortars: Factors affecting the mechanical behavior. Cem. Concr. Res..

[56] (1999). EN 1015-3, Methods of Test for Mortar for Masonry—Part 3: Determination of Consistence of Fresh Mortar (by Flow Table).

[57] (1998). EN 1015-2, Methods of Test for Mortar for Masonry—Part 2: Bulk Sampling of Mortars and Preparation of Test Mortars.

[58] (2016). EN 196-1, Methods of Testing Cement—Part 1: Determination of Strength.

[59] (2010). EN 196-6, Methods of Testing Cement—Part 6: Determination of Fineness.

[60] (2012). EN 933-1, Testing for Geometrical Properties of Aggregates–Part 1: Determination of Particle Size Distribution.

[61] (2013). EN 1097-6, Tests for Mechanical and Physical Properties of Aggregates–Part 6: Determination of Particle Density and Water Absorption.

[62] (2009). NF P 18-513, 2009. Pozzolanic Addition for Concrete-Metakaolin: Definitions, Specifications and Conformity Criteria, Annex A.

[63] Záleská M., Pavlíková M., Pavlík Z., Jankovský O., Pokorný J., Tydlitát V., Svora P., Černý R. (2018). Physical and chemical characterization of technogenic pozzolans for the application in blended cements. Constr. Build. Mater..

[64] Pavlíková M., Zemanová L., Pokorný J., Záleská M., Jankovský O., Lojka M., Sedmidubský D., Pavlík Z. (2018). Valorization of wood chips ash as an eco-friendly mineral admixture in mortar mix design. Waste Manag..

[65] (1999). EN 1015-10, Methods of Test for Mortar for Masonry—Part 10: Determination of Dry Bulk Density of Hardened Mortar.

[66] (1999). EN 1015-11, Methods of Test for Mortar for Masonry-Part 10: Determination of Flexural and Compressive Strength of Hardened Mortar.

[67] (2002). EN 1015-18, Methods of Test for Mortar for Masonry—Part 18: Determination of Water-Absorption Coefficient Due to Capillary Action of Hardened Mortar.

[68] Kumaran M.K. (1999). Moisture diffusivity of building materials from water absorption measurements. J. Therm. Envel. Build. Sci..

[69] Pavlík Z., Černý R. (2012). Determination of moisture diffusivity as a function of both moisture and temperature. Int. J. Thermophys..

[70] (2001). ISO 12572, Hygrothermal Performance of Building Materials and Products—Determination of Water Vapour Transmission Properties.

[71] Záleská M., Pavlík Z., Čítek D., Jankovský O., Pavlíková M. (2019). Eco-friendly concrete with scrap-tyre-rubber-based aggregate–Properties and thermal stability. Constr. Build. Mater..

[72] Luo K., Li J., Lu Z.Y., Jiang J., Niu Y.H. (2019). Effect of nano-SiO_2_ on early hydration of natural hydraulic lime. Constr. Build. Mater..

[73] Lea F.M. (1976). The Chemistry of Cement and Concrete.

[74] Grilo J., Santos Silva A., Faria P., Gameiro A., Veiga R., Velosa A. (2014). Mechanical and mineralogical properties of natural hydraulic lime-metakaolin mortars in different curing conditions. Constr. Build. Mater..

[75] Raverdy M., Brivot F., Paillére A.M., Dron R. Appréciation de I’Activité Pouzzolanique des Constituants Secondaires. Proceedings of the 7th International Congress on the Chemistry of Cement.

[76] Le Bas M.J., Le Maitre R.W., Streckeisen A., Zanettin B. (1986). A chemical classification of volcanic rocks based on the total alkali-silica diagram. J. Petrol..

[77] Wakizaka Y. (2000). Alkali–silica reactivity of Japanese rocks. Eng. Geol..

[78] Jozwiak-Niedzwiedzka D., Antolik A., Dziedzic K., Glinicki M.A., Gibas K. (2019). Resistance of selected aggregates from igneous rocks to alkali-silica reaction: Verification. Roads Bridges Drogi I Mosty.

[79] Tapan M. (2014). Alkali–silica reactivity of alkali volcanic rocks. Eur. J. Environ. Civ. Eng..

[80] Garijo L., Azenha M., Ramesh M., Lourenço P.B., Ruiz G. (2019). Stiffness evolution of natural hydraulic lime mortars at early ages measured through EMM-ARM. Constr. Build. Mater..

[81] Pachta V., Triantafyllaki S., Stefanidou M. (2018). Performance of lime-based mortars at elevated temperatures. Constr. Build. Mater..

[82] Palomar I., Barluenga G., Puentes J. (2015). Lime-cement mortars for coating with improved thermal and acoustic performance. Constr. Build. Mater..

[83] Ramesh A., Ayenha M., Lourenço P.B. (2019). Mechanical properties of lime-cement masonry mortars in their early ages. Mater. Struct..

[84] Singhal V., Rai D.C. (2014). Suitability of half-scale burnt clay bricks for shake table tests on masonry walls. J. Mater. Civ. Eng..

[85] Veiga M., Velosa A., Magalhães A. (2007). Evaluation of mechanical compatibility of renders to apply on old walls based on a restrained shrinkage test. Mater. Struct..

[86] (2016). EN 998-1, Specification for Mortar for Masonry—Part 1: Rendering and Plastering Mortar.

[87] Nogueira R., Ferreira Pinto A.P., Gomes A. (2018). Design and behaviour of traditional lime-based plasters and renders. Review and critical appraisal of strengths and weaknesses. Cem. Concr. Compos..

[88] Pavlíková M., Zemanová L., Záleská M., Pokorný J., Lojka M., Jankovský O., Pavlík Z. (2019). Ternary blended binder for production of a novel type of lightweight repair mortar. Materials.

[89] Fusade L., Viles H., Wood C., Burns C. (2019). The effect of wood ash on the properties and durability of lime mortar for repointing damp historic buildings. Constr. Build. Mater..

[90] Roels S., Carmeliet J., Hens H., Adan O., Brocken H., Cerny R., Pavlik Z., Hall C., Kumaran K., Pel L. (2004). Interlaboratory comparison of hygric properties of porous building materials. J. Therm. Envel. Build. Sci..

[91] Chennouf N., Agoudjil B., Boudenne A., Benzarti K., Bouras F. (2018). Hygrothermal characterization of a new bio-based construction material: Concrete reinforced with date palm fibers. Constr. Build. Mater..

[92] Silva B.A., Ferreira Pinto A.P., Gomes A. (2014). Influence of natural hydraulic lime content on the properties of aerial lime-based mortars. Constr. Build. Mater..

[93] Bianco N., Calia A., Denotarpietro G., Negro P. (2013). Hydraulic mortar and problems related to the suitability for restoration. Period. Miner..

[94] Ochs F., Heidemann W., Mueller-Steinhagen H. (2008). Effective thermal conductivity of moistened insulation materials as a function of temperature. Int. J. Heat Mass Transf..

[95] Wang Y., Zhao Z., Liu Y., Wang D., Ma C., Liu J. (2019). Comprehensive correction of thermal conductivity of moist porous building materials with static moisture distribution and moisture transfer. Energy.

[96] Lide D.R. (1998). CRC Handbook of Chemistry and Physics.

[97] (2012). EN 1745, Masonry and Masonry Products—Methods for Determining Thermal Properties.

[98] Palomar I., Barluenga G. (2017). Assessment of lime-cement mortar microstructure and properties by P- and S- ultrasonic waves. Constr. Build. Mater..

